# The effect of COVID-19 process on patients with endocrinological disease in a pandemic hospital: What happened to the others?

**DOI:** 10.20945/2359-3997000000525

**Published:** 2022-10-11

**Authors:** Evin Bozkur, Seda Turgut, Naim Pamuk, Hamide Piskinpasa, Duygu Metin, Ahmet Cem Dural, Nuri Alper Sahbaz, Omur Gunaldi, İlkay Cakir, Meral Mert, Sema Ciftci Dogansen

**Affiliations:** 1 University of Health Sciences Bakirkoy Dr. Sadi Konuk Training and Research Hospital Department of Endocrinology and Metabolism Istanbul Turkey University of Health Sciences, Bakirkoy Dr. Sadi Konuk Training and Research Hospital, Department of Endocrinology and Metabolism, Istanbul, Turkey; 2 University of Health Sciences Bakirkoy Dr. Sadi Konuk Training and Research Hospital Department of Radiology Istanbul Turkey University of Health Sciences, Bakirkoy Dr. Sadi Konuk Training and Research Hospital, Department of Radiology, Istanbul, Turkey; 3 University of Health Sciences Bakirkoy Dr. Sadi Konuk Training and Research Hospital Department of Surgery Istanbul Turkey University of Health Sciences, Bakirkoy Dr. Sadi Konuk Training and Research Hospital, Department of Surgery, Istanbul, Turkey; 4 University of Health Sciences Bakirkoy Prof. Dr. Mazhar Osman Training and Research Hospital for Neurology, Neurosurgery and Psychiatry Department of Neurosurgery Istanbul Turkey University of Health Sciences, Bakirkoy Prof. Dr. Mazhar Osman Training and Research Hospital for Neurology, Neurosurgery and Psychiatry, Department of Neurosurgery, Istanbul, Turkey

**Keywords:** Adrenal, COVID-19 pandemic, endocrinology, pituitary, thyroid

## Abstract

**Objective:**

To evaluate the effects of the pandemic process on those with an endocrinological disease that will require close follow-up from the last visit before the pandemic.

**Materials and methods:**

Patients of 3,903 with thyroid, calcium-bone metabolism, adrenal gland, pituitary diseases, and neuroendocrine tumor (NET) were retrospectively scanned. The remaining 855 (656 females and 199 males) patients with active disease or who still needed multidisciplinary approaches were included. The number of patients who continued the disease-related medical procedures and could complete these procedures on time in the pandemic period was determined, and medical deprivation rate (MDR) was calculated.

**Results:**

The prepandemic period of our patients with thyroid disease (n = 594), calcium-bone metabolism disorder (n = 130), adrenal disease (n = 85), pituitary disease, and NET (n = 46) had MDRs of 85%, 56%, 81%, and 89%, respectively. For each subgroup of patients, the lowest MDR (67%) was in medullary thyroid carcinoma, the highest MDR (89%) was in differentiated thyroid carcinoma; the lowest MDR (6%) was in osteoporosis, the highest MDR (100%) was in the active Paget's disease; the lowest MDR (0%) was in primary adrenocortical insufficiency, the highest MDR (100%) was in hyperfunctional adrenal adenomas; the lowest MDR (81%) was in pituitary nonfunctional adenomas, and the highest MDR (100%) was in Cushing's disease, active prolactinoma, TSHoma, and NET, respectively.

**Conclusion:**

This study showed that not only those who had COVID-19 but also those who had medical deprivation due to their current endocrinological disease were not to be underestimated during the pandemic period.

## INTRODUCTION

COVID-19 is a disease caused by severe acute respiratory syndrome coronavirus 2 (SARS-CoV-2), which was first seen in China and spread rapidly worldwide. The World Health Organization declared the outbreak, and it has been devastating in many countries. Its effects on people continue (1–3). Furthermore, the pandemic affected not only those with COVID-19 but also many other patients with chronic diseases. Patients did not admit to hospitals for fear of being infected, many medical centers were declared as pandemic hospitals, physicians mostly started working in COVID-19 clinics regardless of their expertise, and outpatient admissions were suspended. These factors led to a state of medical deprivation due to pandemic conditions, such as quarantine rules, pandemic lockdown, changes in the hospital working order, or fear of COVID-19, independent of financial reasons. The relevant medical societies published declarations that elective interventional procedures and elective during this period as well as the imaging tests being mainly used for patients with COVID-19 were among the factors that affected patients with chronic diseases (4–10).

Patients with the endocrinological disease have a better general condition than do many other patients with internal diseases, and outpatient clinics are more prominent than inpatient clinics are in endocrinology. This group of patients has a state of well-being that leads them to avoid hospitals during the pandemic period. A study conducted in our country demonstrated that the most significant decrease in the number of patients was detected in outpatient endocrine surgery clinics (11). Moreover, the evaluation of patients in endocrinology often requires many dynamic tests and radiological assessments in addition to physical and hormonal examinations. Multidisciplinary teams, including general surgeons, radiologists, pathologists, nuclear medicine doctors, and neurosurgeons, also often provide the conclusion of cases (4,12).

Even though it has been a short time since the outbreak of the COVID-19 pandemic, many studies, reviews, and even meta-analyses have been published due to it being a critical global problem (1,2,13,14). However, almost all these studies have been about infected individuals or the risk of infection susceptibility. These studies are a priority for understanding the disease with many unknowns. On the other hand, the effects of postponing other medical services due to the pandemic on these relatively stable patients and the course of diseases in this period are also significant. To the best of our knowledge, no study on the medical deprivation of endocrinology patients due to pandemic. Therefore, this study aimed to evaluate the course of the disease and medical deprivation in endocrinology patients.

## MATERIALS AND METHODS

This study is a retrospective study. The first COVID-19 case in Turkey was reported on on 11 March 2020 (15), and this study was designed considering that date. Therefore, 1 January 2020 and 11 March 2020 was accepted as the prepandemic period. Our endocrine center is a tertiary center that provides both an inpatient clinic and four active outpatient clinics working with a remote appointment system for each day. Our endocrine surgery clinic is a center with 14 operation days per month and an average of 3-4 surgeries per day. The pituitary surgery clinic we work with is a center with an average of 12 operation days per month and an average of 2-3 pituitary surgeries per day. In our interventional radiology center, an average of 80 thyroid fine-needle aspirations (FNAs) could be performed weekly, and adrenal venous sampling (AVS), inferior petrosal sinus sampling (IPSS), and parathyroid venous sampling are performed if there are cases.

**Figure 1:**
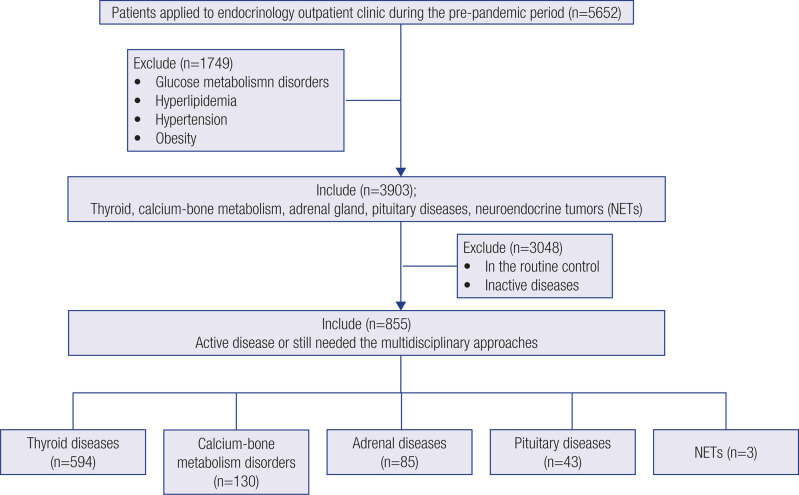
Flowchart of the enrollment process

A few days after the first case was reported in Turkey, our center began accepting patients with COVID-19 mainly because the center was designated a pandemic hospital. A new working system began, in which patients with endocrinological diseases were examined only in outpatient clinics and consultations. Due to the precautions taken in our country, patients with chronic diseases could take their medicines from the pharmacies without a prescription in this period. There was no change in the existing health insurance of all our patients who were under the comprehensive health care policies in our country, during the 3-month pandemic period. Elective surgeries and radiological interventions were postponed approximately one week after the first case was reported, and interventional procedures were performed only in emergency cases. This period lasted until the number of patients with COVID-19 stabilized, approximately 2.5 months up to 1 June 2020. This duration was called the pandemic period in our study. All of these factors meant patients in this study could not apply to our outpatient clinic for their follow-up examinations and ongoing medical procedures; in other words, the main cause of “medical deprivation” was accepted as the pandemic conditions who applied to our endocrinology outpatient clinics during the prepandemic period. Patients with diseases associated with COVID-19, such as glucose metabolism disorders, hyperlipidemia, hypertension, or obesity, were excluded, and 3,903 remaining patients with thyroid, calcium-bone metabolism, adrenal gland, pituitary diseases, and neuroendocrine tumor (NET) were obtained. Patients were in a routine control, or those whose inactive in the prepandemic period were excluded. The remaining 855 (656 females and 199 males) patients with active disease or who still needed the endocrinological or multidisciplinary approaches were included in the study. A flowchart of the enrollment process, inclusion, and exclusion criteria was shown in Figure 1.

Those who applied with thyroid diseases in the prepandemic period were recorded as follows: patients with overt hypothyroidism (low fT4 and TSH > 10 mU/L) and overt thyrotoxicosis with levothyroxine (TSH < 0.1mU/L and high fT4); patients with overt hyperthyroidism, antithyroid drug (ATD) side effects, and planned FNAs, surgery, or radioactive iodine (RAI) therapy; patients with both Graves’ disease and toxic nodular goiter; patients with active subacute thyroiditis; and patients with euthyroid nodular goiter planned for FNA or surgery. The reasons for planned surgery were as follow: suspicious cytology (Bethesda 1 or 3 at least twice and Bethesda 4 at least once), malignant cytology (Bethesda 5 and 6), and large nodule or intrathoracic goiter. Patients with differentiated thyroid carcinoma (DTC) and medullary thyroid carcinoma (MTC) were recorded as follows: newly diagnosed, needed only follow-up, resurgery, RAI, peptide-receptor radionuclide therapy with ^177^Lu, and treatment with a tyrosine kinase inhibitor.

Those who applied with calcium-bone metabolism disorders in the prepandemic period were recorded as follows: hypoparathyroidism patients with hypocalcemia (albumin corrected calcium < 8 mg/dL); primary hyperparathyroidism patients with hypercalcemia (albumin corrected calcium > 12 mg/dL) and preoperative preparations who were planned for surgery; patients with active Paget's disease who were followed up with further investigation or were planned to receive parenteral bisphosphonates; patients with osteoporosis who received denosumab or teriparatide and were planned for treatment with zoledronic acid.

Patients with adrenal diseases who applied in the prepandemic period were recorded as follows: patients with newly diagnosed primary adrenocortical insufficiency (AI) and patients who were followed up with further examination/treatment or were planned for surgery, from those with nonfunctional adrenal adenoma, functional adrenal adenoma, pheochromocytoma, and paraganglioma.

The patients with functional or nonfunctional pituitary adenoma were recorded as follows: patients who were followed up with further examinations and planned for stereotactic radiosurgery or surgery or patients whose disease was still active at the last visit in the prepandemic period. The status of patients with NET at the last visit was evaluated.

The number of patients who applied in the prepandemic period who also came during the pandemic period and their number of disease-related medical procedures (DRMPs; *e.g.*, IPSS, FNA, AVS, diagnostic tests, medical treatments, RAI treatment, and surgery) that could be completed on time in the pandemic period were determined. Thus, the medical deprivation rate (MDR) was calculated accordingly. The MDR formula was calculated as follows for each disease group: 100 × (the number of patients deprived of medical service because they could not complete the DRMP in the pandemic period / the number of patients who still needed endocrinological or multidisciplinary approaches in the prepandemic period).

The local ethics committee approved this study of the University of Health Sciences, Bakırköy, Dr. Sadi Konuk Training and Research Hospital, and all procedures were conducted in accordance with the Helsinki Declaration.

SPSS Version 20.0 software was used for statistical analysis. Only descriptive variables were presented as frequency and ratio.

## RESULTS

The prevalence of patients who did not complete their DRMPs in the prepandemic period was 22% (855 of 3903). Twenty-two percent of patients (191 of 855) applied to the endocrinology outpatient clinic during the prepandemic period. However, only 19% (165 of 855) of patients could complete the DRMP during the pandemic period. In other words, MDR reached 81% in patients with an endocrinological disease who required close follow-up during the pandemic period.

Detailed data on the courses of those with any thyroid disease requiring follow-up since the last visit of the prepandemic period are summarized in Table 1. Accordingly, only 11% (3 of 27) of our patients with overt hypothyroidism or thyrotoxicosis with levothyroxine completed DRMP; the MDR of this group was 89%. Fifty percent (2 of 4) of those with active subacute thyroiditis completed DRMP; the calculated MDR was 50%.

**Table 1:** The course of those with any thyroid disease requiring follow-up since the last visit of the pre-pandemic period

N = 594 (461 F/133 M)		Applied to hospital in Pandemic Period (n; %)	Completed DRMP in Pandemic Period (n; %)
Patients with hypothyroidism (n = 27; 21 F/6 M)	Overt hypothyroidism (n = 15)	2 (13)	2 (13)
	Thyrotoxicosis with LT4 (n = 12)	1 (8)	1 (8)
Patients with Graves’ disease (n = 141; 105 F/36 M)	Overt hyperthyroidism (n = 112)	32 (29)	16 (14)
	ATD side effect (n = 2)	2 (100)	1 (50)
	Planned FNA (n = 11)	3 (27)	3 (27)
	Planned surgery (n = 22)	4 (18)	2 (9)
Patients with toxic nodular goiter (n = 164; 121 F/43 M)	Overt hyperthyroidism (n = 66)	10 (15)	9 (14)
	ATD side effect (n = 0)	–	–
	Planned FNA (n = 70)	12 (17)	12 (17)
	Planned surgery (n = 37)	9 (24)	2 (5)
	Planned RAI n = 3)	1 (33)	–
Patients with active subacute thyroiditis (n = 4; 2 F/2 M)	Glucocorticoid therapy (n = 0)	–	–
	Symptomatic therapy (n = 4)	2 (50)	2 (50)
Patients with euthyroid nodular goiter (n = 215; 183 F/32 M)	Planned FNA (n = 149)	17(11)	17 (11)
	Planned surgery (n = 66)	8 (12)	7 (11)
	a. Suspicious cytology (n = 41)	2 (5)	1 (2)
	b. Malign cytology (n = 14)	4 (29)	4 (29)
	c. Large nodule size or intrathoracic goiter (n = 11)	2 (18)	2 (18)
Patients with new diagnosis or non-remission DTC (n = 37; 27 F/10 M)	Only TSH suppressive treatment (n = 15)	4 (27)	3 (20)
	Requiring additional interventions (n = 23)		
	a. Planned resurgery (n = 0)	–	–
	b. Planned RAI (n = 23)	1 (4)	1 (4)
Patients with new diagnosis or non-remission MTC (n = 6; 2 F/4 M))	New diagnosis with normal calcitonin levels (n = 1)	1 (100)	1 (100)
	High calcitonin levels (n = 5)		
	Planned resurgery (n = 1)		
	PRRT with ^177^ Lu and tyrosine kinase inhibitor (n = 1)	–	–
	Only follow-up (n = 3)	1 (33)	1 (33)

DRMP: disease-related medical procedures; ATD: anti-thyroid drug; DTC: differentiated thyroid carcinoma; FNA: fine-needle aspiration; MTC: medullary thyroid carcinoma; PRRT: peptide receptor radionuclide therapy; RAI: radioactive iodine.

Since the last follow-up visit of patients with Graves’ disease, 16% (22 of 141) of those with hyperthyroidism, ATD side effects, and planned for FNA or surgery completed DRMP; the calculated MDR was 84%. Of patients who were followed up with for toxic nodular goiter at the last visit, 14% (23 of 164) of the patients with hyperthyroidism, ATD side effects, planned for FNA, surgery, or RAI treatment completed DRMP; the MDR was 86%. Fourteen percent (31 of 215) of the patients with euthyroid nodular goiter planned for FNA or surgery completed DRMP; the calculated MDR was 86%.

Eleven percent (4 of 37) of patients with nonremission or newly diagnosed DTC, those with follow-up for TSH suppression, and those planned for resurgery or RAI completed DRMP; the MDR was 89%. One patient with newly diagnosed MTC with normal calcitonin levels and those who were following up with high calcitonin levels (*n* = 5) were admitted to the outpatient clinic during the prepandemic period. Only 33% (2 of 6) of patients completed DRMP, and the MDR was 67%.

The course of patients with any calcium-bone metabolism disorders requiring follow-up since the last visit of the prepandemic period was summarized in Table 2. Accordingly, 25% (5 of 20) of hypoparathyroidic patients with hypocalcemia completed DRMP, and the MDR was 75%. Among the hypercalcemic patients followed with hyperparathyroidism, only 2% (1 of 51) of those with continued localization studies or planned surgery completed DRMP, and the calculated MDR was 98%. None of the patients with active Paget's disease could complete DRMP, and the MDR was 100%. Ninety-four percent (51 of 55) of osteoporosis patients who received zoledronic acid, denosumab, or teriparatide therapy were able to receive their treatment; MDR was 6%.

**Table 2:** The course of patients with any calcium-bone metabolism disorders requiring follow-up since the last visit of the pre-pandemic period

N = 130 (116 F/14 M)		Applied to hospital in Pandemic Period (n; %)	Completed DRMP in Pandemic Period (n; %)
Hypoparathyroidic patients with hypocalcemia (n = 20; 17 F/3 M)		5 (25)	5 (25)
Patients with primary hyperparathyroidism (n = 51; 45 F/6 M)	Overt hypercalcemia (n = 5)	5 (100)	–
	Preop preparation (n = 20)	7 (35)	–
	Planned surgery (n = 31)	4 (13)	1 (3)
Patients with active Paget's disease (n = 4; 3F/1 M)	Further investigation (n = 3)	1 (33)	–
	Planned parenteral biphosphonat (n = 1)	–	–
Osteoporosis patients with parenteral therapy (n = 55; 51 F/4 M)	Planned parenteral zoledronic acid (n = 4)	–	–
	Denosumab (n = 19)	2 (11)	19 (100)
	Teriparetide (n = 32)	4 (13)	32 (100)

DRMP: disease-related medical procedures.

Detailed data on the course of patients with an adrenal disease that required follow-up since the last visit of the prepandemic period were shown in Table 3. Accordingly, two patients who were diagnosed with primary AI in the prepandemic period applied to the outpatient clinic in the pandemic period, and their complete medications were provided; therefore, they had no medical deprivation. Twenty-two percent (14 of 63) of patients who were followed up with further investigation or planned surgery of those with adrenal incidentaloma or nonfunctional adenomas completed DRMP; MDR was 78%. None of 5 adrenal CS patients with planned surgery could be operated on, and the MDR was 100%. None of 11 patients with hyperaldosteronism awaiting verification tests, AVS, or surgery completed DRMP, and the MDR was 100%. None of 4 patients followed up with pheochromocytoma or paraganglioma completed DRMP, and the MDR rate was 100%.

**Table 3:** The course of patients with an adrenal disease requiring follow-up since the last visit of the pre-pandemic period

N = 85 (59 F / 26 M)		Applied to hospital in Pandemic Period (n; %)	Completed DRMP in Pandemic Period (n; %)
Patients with newly diagnosed primary AI (n = 2; 2 F)		2 (100)	2 (100)
Patients with adrenal incidentaloma or non-functional adenomas (n = 63; 44 F/19 M)	Further investigation (n = 53)	19 (36)	10 (19)
	Planned surgery (n = 10)	5 (50)	4 (40)
Patients with adrenal CS (n = 5; 5 F)	Planned surgery for SCS (n = 1)	–	–
	Planned surgery for severe CS (n = 4)	2 (50)	–
Patients with hyperaldosteronism (n = 11; 6 F/5 M)	Awaiting verification test (n = 6)	1 (17)	–
	Planned AVS (n = 2)	1 (50)	–
	Planned surgery (n = 3)	–	–
Patients with pheochromocytoma or paraganglioma (n = 4; 2 F/2 M)	Further investigation/treatment (n = 1)	–	–
	Planned surgery (n = 3)	1 (33)	–

DRMP: disease-related medical procedures; AI: adrenal insufficiency; AVS: adrenal venous sampling; CS: Cushing's syndrome; SCS: subclinic Cushing's syndrome.

Detailed data of patients with any pituitary disease and NETs requiring follow-up since the last visit during the prepandemic period were summarized in Table 4. Nineteen percent (3 of 16) of patients with incidental or nonfunctional adenoma who continued to be examined or were planned for stereotactic radiosurgery or surgery completed DRMP, and the MDR was 81%. In this period, none of those with active Cushing’ disease completed DRMP, and MDR was 100%. Fifteen percent (2 of 13) of patients with active acromegaly completed DRMP, and MDR was 85%. Neither patients with active prolactinomas (*n* = 5) nor patients with active TSHoma (*n* = 1) completed DRMP, and MDR was 100% for both. One of 3 patients diagnosed with NET applied, and none completed DRMP; MDR was 100%.

**Table 4:** The course of patients with any pituitary disease and neuroendocrine tumors requiring follow-up since the last visit of the pre-pandemic period

N = 46 (20 F/26 M)		Applied to hospital in Pandemic Period (n; %)	Completed DRMP in Pandemic Period (n; %)
Patients with incidental or non-functional adenomas (n = 16; 5 F, 11 M)	Planned surgery (n = 3)	1 (33)	–
	Planned SRS (n = 0)	–	–
	Further investigation (n = 13)	5 (38)	3 (23)
Patients with active Cushing's disease (n = 8; 5 F, 3 M)	Planned surgery (n = 5)	–	
	Planned SRS (n = 1)	1 (100)	–
	Further investigation or follow-up with medical treatment (n = 2)	1 (50)	–
Patients with active acromegaly (n = 13; 5 F, 8 M)	Planned surgery (n = 2)	1 (50)	–
	Planned SRS (n = 1)	1 (100)	1 (100)
	Further investigation or follow-up with medical treatment (n = 10)	2 (20)	1 (10)
Patients with active prolactinoma (n = 5; 1 F, 4 M)	Follow-up with medical treatment (n = 5)	1 (20)	–
Patients with active TSHoma (n = 1)	Follow-up with medical treatment (n = 1)	–	–
Patients with active NET (n = 3; 3 F)	Further investigation (n = 2)	1 (50)	–
	Follow-up with medical treatment (n = 1)	–	–

DRMP: disease-related medical procedures; NET: neuroendocrine tumor; SRS: stereotactic radiosurgery.

None of the patients died due to COVID-19 or other causes, including during the specified period for the study.

## DISCUSSION

This study has indicated that those who had COVID-19 and those who had medical deprivation due to their current endocrinological disease were notable in the pandemic. With the decrease in the number of COVID-19 cases in countries affected by the pandemic, other patients affected by medical deprivation will become more prominent. The COVID-19 pandemic was able to be controlled earlier in Turkey than it was in many countries (16). Moreover, this allowed us to show the effects of the pandemic on our patients earlier, and to the best of our knowledge, this is the first study in the field of endocrinology.

Patients with thyroid diseases constituted the majority of the population who applied to the outpatient clinic. While MDRs were quite different in subgroups of thyroid diseases, most hyperthyroidism patients could not achieve euthyroidism in the pandemic period. Clinical information and comment from the European Society of Endocrinology in the time of COVID-19 suggested that those who are well controlled by titration should continue. However, a block-and-replace regimen (BRR) was recommended in newly diagnosed or relapsed/active Graves’ disease during this period (17). Because BRR has similar effectiveness in providing euthyroidism compared to the titration method, this regimen requires less follow-up and testing than titration does (18,19). However, BRR is a treatment used in a minimal number of patients in our country, and we did not have any patients followed up with this regimen. In terms of ATD complications, one patient had a minor side effect (urticarial), while the other had hepatotoxicity. This Graves’ disease patient's medication was discontinued and urgent surgery was planned. However, the patient was able to admit at the end of the pandemic period with increased complaints, and non-ATD treatment was started for the patient with overt hyperthyroidism, then preparations of surgery were initiated. Because no patients had more severe eye involvement than moderate Graves’ orbitopathy in the prepandemic period, none of our patients received steroids, consistent with Graves’ orbitopathy guidelines, local treatments (*e.g.*, artificial tears), protective approaches (*e.g.*, smoking avoidance), and protection of euthyroidism were applied (20). It has been recommended that postponing steroid therapy in cases where Graves’ orbitopathy is not severe and steroids could be given weekly only if it is necessary, or daily doses can be applied to reduce the hospital contact (21). Our patients, who were followed up with active subacute thyroiditis during the prepandemic period, were under symptomatic treatment; none of them received steroid treatment, and 50% were able to apply to the outpatient clinic during the pandemic period. While there is no suggestion for follow-up during the pandemic period, subacute thyroiditis after SARS-COV-2 infection has been reported in only on case in the literature (22). Only 13% of patients with severe hypothyroidism applied during the pandemic period. Experts suggested for this period that patients with hypothyroidism should admit to the hospital if they did not feel well or if they have significant findings such as severe weight changes, etc., rather than follow-up with regular blood tests (17).

FNA-related medical deprivation of thyroid patients was frequent. It is known that less than 10% of them are malignant, although thyroid nodules are quite frequent (23). Moreover, patients with suspicious cytology with FNA were mostly deprived of medical services, and only a few were operated. However, because a few months of delays in diagnosing thyroid cancer do not have noticeable adverse effects on the course of the disease, postponing surgeries and procedures such as ultrasonography, FNA, and scintigraphy for asymptomatic thyroid nodules was recommended in the pandemic period (24). However, MTC is the exception, and DRMP should be completed even during the pandemic period, but there were no patients with suspected MTC in the prepandemic period in our series.

In patients with DTC, it was suggested that except for high-risk patients as determined by Martinique criteria (25), other patients could be followed up with suppression treatment. Furthermore, adjuvant RAI treatments or remnant ablation can be postponed during this period (24). RAI was planned for 23 of our patients who were followed up with a new diagnosis or nonremission DTC in the prepandemic period, but only one patient could receive it. It is well known that early diagnosis and treatment improve outcomes and affect survival in MTC, which differs from DTC (26). Therefore, performing the necessary procedures as soon as possible was recommended in these patients (24). In our series, none of these procedures was performed in patients who were planned to have resurgery and peptide-receptor radionuclide therapy with ^177^Lu during the prepandemic period, even the patients who did not apply to the hospital during the pandemic period, and calcitonin levels were found significantly increased in their examination thereafter.

The suggestions in the pandemic period to patients with hypoparathyroidism were to increase the calcium replacements when necessary by following the symptoms but not to change empirical doses of calcitriol or alfacalcidol in cases when no examination can be performed (27). Only one-fourth of our patients with hypoparathyroidism, who were hypocalcemic in the prepandemic period, applied in the pandemic period. Normocalcemia was achieved with replacement dose adjustments.

In primary hyperparathyroidism, it was recommended that the patients should be warned for signs and symptoms of hypercalcemia during the pandemic period (27). Fortunately, only five patients had prominent hypercalcemia during the prepandemic period, in which hydration and symptomatic treatments were repeated intermittently, and there were no major complications. Postponing surgery and localization studies, hydration, mobilization, and urgent treatment or follow-up of calcium were also recommended unless calcium exceeds 13 mg/dL (27). While approximately 40% of our patients with hyperparathyroidism continued preoperative procedures, none of them applied to the outpatient clinic during the pandemic period. Only one of 31 patients with planned surgery could be operated.

It was recommended that teriparatide and denosumab should be continued in patients with osteoporosis. Sustaining calcium-vitamin D replacements and delaying zoledronic acid treatment, or continuing with oral bisphosphonate, was also recommended if there were no contraindications (27). Our patients who received denosumab or teriparatide, which we followed up with in the prepandemic period, continued their treatment. Regulations have been made by the health authorities to reach their regular medication even if they do not admit to the hospital. Consistently, the lowest MDR was in these patients. However, none of our four patients who were planned for parenteral zoledronic acid applied during this period, and their treatment was delayed. We did not find any suggestions regarding active Paget's disease for the pandemic period in the literature. In our series, we had four active Paget patients, and none of them completed the DRMP.

In our series, two of the patients we followed up for primary AI were diagnosed during the prepandemic period, and both were examined and treated during the pandemic period. Expert advice on this issue addresses the importance of raising awareness of self-management so that patients can better manage intervening conditions (28). Our patients diagnosed with AI were already given steroid emergency cards and training on this subject in our clinic.

A few patients with adrenal incidentaloma could complete DRMP, whereas 40% of those planned for surgery were operated on due to malignancy risk. In endocrine surgery, experts already recommended urgent adrenalectomy for those diagnosed with adrenocortical carcinoma and those suspicious in this regard (5). Pheochromocytoma and paraganglioma, semiurgent operation, and follow-up with medical treatment were recommended unless patients present with heart failure (5,29). In our series, three patients, one with pheochromocytoma and two with paraganglioma, were only monitored with alpha blockage. Deferred adrenalectomy was recommended for hyperaldosteronism (5). In our series, all procedures of patients waiting for examination or surgery were already delayed. The highest MDRs were in patients with pheochromocytoma, paraganglioma, and hyperaldosteronism due to the recommendations mentioned above.

The patients with the most critical endocrinological diseases in this period were undoubtedly those with Cushing's syndrome (CS). Immunosuppression by hypercortisolemia in these patients leads to a high risk of COVID-19 infection in transmission and disease progression (5,30). The recommendations for these patients were a medical treatment for severe hypercortisolism and semiurgent adrenalectomy in cases with adrenal CS that cannot be controlled by medical approaches (29,30). While none of both patients with adrenal CS and Cushing's disease were operated on during this period, they were followed up with ketoconazole. In our series, we did not perform any procedures, including IPSS, during the pandemic period, for all our patients who were either planned for surgery or were continued for further investigation. Medical treatment was recommended for CS, primarily dependent on the country's conditions regardless of etiology, and gradual dose increases have been suggested for these patients without causing AI (6,7,30,31).

Pituitary society has classified pituitary surgeries into elective, urgent, and emergent categories during the pandemic period (6,7). Accordingly, masses with pituitary apoplexy, severe vision loss, increased pressure effect, and malignant pathologies were considered emergent. Our series had a patient with ptosis due to cavernous sinus invasion, but he could not be operated on during the pandemic period because he was elderly and his relatives did not accept the procedure. On the other hand, it was accepted as an urgent condition, but no patients had urgent conditions in our series. According to expert suggestions, elective cases could be monitored with or without medical treatment (6,7).

In the prepandemic period, those who had an active disease (*e.g.*, cabergoline and somatostatin analog), but received medical treatment, continued their medications. Most of our patients who were followed up with for further investigation did not apply to the outpatient clinic. only paying attention to hypopituitarism or diabetes insipidus in this period and trying to complete the DRMP for dynamic tests without bringing the patient to the hospital multiple times and evaluating the hormone hypersecretions after the pandemic was recommended (30,32).

In our series, one patient with an operated pancreatic NET under somatostatin analog and two patients with ectopic ACTH syndrome (EAS) and whose localization tests were lasting were followed up with ketoconazole. During the pandemic period, if EAS etiology was a well-differentiated NET, then a delay of 3-6 months would not affect the patient's survival so that the DRMP could be delayed (30). Because hypercortisolemia will affect the actual prognosis rather than the mass effect, medical treatment was recommended according to country availability (30). We preferred the ketoconazole that we can reach regarding country availability.

In conclusion, the MDRs that we found were quite high in endocrinological patients during the pandemic period, although a social health policy provides comprehensive health insurance for all citizens in Turkey. While this is the case in endocrine patients with a better health condition, medical deprivation rates should be determined in patients with more serious chronic diseases by extensive studies that each country may also test its health system. Additionally, this study revealed the consequences of medical deprivation for patients, pointing to the possible importance of remote monitoring systems for patients and highlighting the need for further studies in this regard.
